# Normal tissue complication probability modeling of severe radiation-induced lymphopenia using blood dose for lung cancer patients treated with IMRT and IMPT

**DOI:** 10.3389/fimmu.2026.1752785

**Published:** 2026-04-07

**Authors:** Shuting Wang, Tianyuan Dai, Xiaoying Fan, Weijie Li, Wei Huang, Ruozheng Wang, Yong Yin

**Affiliations:** 1 Department of Graduate, Shandong First Medical University, Shandong Academy of Medical Sciences, Jinan, China; 2 Department of Radiation Oncology Physics and Technology, Shandong Cancer Hospital and Institute, Shandong First Medical University and Shandong Academy of Medical Sciences, Jinan, China; 3 College of Intelligence and Information Engineering, Shandong University of Traditional Chinese Medicine, Jinan, China; 4 Department of Radiation Oncology, Shandong Cancer Hospital and Institute, Shandong First Medical University and Shandong Academy of Medical Sciences, Jinan, China; 5 Department of Radiation Oncology, The Third Affiliated Teaching Hospital of Xinjiang Medical University, Affiliated Cancer Hospital, Urumqi, China

**Keywords:** lung cancer, Lyman model, lymphopenia, normal tissue complication probability, radiation-induced toxicity

## Abstract

**Background:**

Severe radiation-induced lymphopenia (SRIL) is a poor prognostic factor in lung cancer. This study aimed to develop and validate normal tissue complication probability (NTCP) models for SRIL based on hematologic dose in patients receiving photon and proton radiotherapy.

**Methods and materials:**

We retrospectively analyzed 131 lung cancer patients receiving curative-intent radiotherapy (94 with IMRT, 37 with IMPT) between 2022 and 2025. Whole-body blood dose-volume histograms were calculated by using the HEDOS framework. The Lyman-Kutcher-Burman NTCP model was adopted, with parameters optimized by maximum likelihood estimation. Model performance was assessed using the area under the receiver operating characteristic curve (AUC) and Brier score.

**Results:**

The incidence of SRIL was 61.7% and 32.4% in the IMRT and IMPT cohorts, respectively. Blood generalized equivalent uniform dose was an independent predictor of SRIL in the IMRT cohort (OR = 4.682, p=0.002). The NTCP model demonstrated strong predictive power in both cohorts (IMRT: AUC = 0.82; IMPT: AUC = 0.80) Model parameters differed between modalities, particularly the volume-effect parameter (a = 19.85 for IMRT *vs*. 2.35 for IMPT). Cross-modality validation of the IMRT-derived model in the IMPT cohort revealed suboptimal calibration (calibration slope=0.54), indicating systematic overestimation of risk and reflecting the distinct blood dose distributions and parameter covariance between modalities.

**Conclusion:**

Modality-specific NTCP models are required to accurately predict SRIL in lung cancer radiotherapy. The IMRT- and IMPT-based models developed in this study demonstrated strong performance in their respective cohorts. Differences in model parameters underscore the influence of modality-dependent blood dose distributions, supporting the development of separate risk models for photon and proton therapy.

## 1 Introduction

Lung cancer is one of the most prevalent malignancies and remains the leading cause of cancer-related morbidity and mortality worldwide (1, 2). Current treatment modalities encompass surgery, radiotherapy, chemotherapy, and, more recently, molecularly targeted therapies and immunotherapy (3–5). Despite advances in multimodal management, the clinical prognosis of lung cancer remains unsatisfactory. In particular, the 5-year overall survival (OS) rate for patients with non-metastatic SCLC and NSCLC is still dismal, ranging between 15% and 30% (6, 7), underscoring the urgent need for improved therapeutic strategies and optimized treatment paradigms.

The immune system plays a pivotal role in tumor surveillance and antitumor defense (8–10). As a central component of the immune system, lymphocytes contribute to both adaptive and innate immune responses and are capable of directly mediating tumor cell destruction (11–13). Nevertheless, radiotherapy, a cornerstone in the treatment of lung cancer, while effectively eradicating malignant cells, simultaneously induces substantial collateral damage to circulating lymphocytes. A recent meta-analysis encompassing 14 studies reported that the average incidence of severe lymphopenia—defined as an absolute lymphocyte count (ALC)< 500/μL—was as high as 64.2% among lung cancer patients undergoing radiotherapy (14). Furthermore, accumulating evidence indicates that the incidence and severity of treatment-related lymphopenia are influenced by multiple factors, including the administration of chemotherapy, treatment duration, irradiated field size, and the radiation dose delivered to critical organs such as the lung, heart, or the body as a whole (15–20). Importantly, lymphopenia has been increasingly recognized as a negative prognostic factor in lung cancer (17, 21–23).

Accurate quantification of the radiation dose delivered to circulating lymphocytes during radiotherapy is essential for understanding the biological mechanisms underlying radiation-induced lymphopenia and for developing reliable predictive models. Conventional models rely on fixed anatomical structures for dose calculation (24–26), failing to account for the dynamic circulation of lymphocytes through the vascular system and multiple organs, which leads to an inaccurate quantification of actual radiation exposure to immune cells. To address this limitation, several modeling approaches have been proposed to estimate radiation dose to circulating blood (27–29). The recently developed hematological dose (HEDOS) framework (30) enables precise calculation of cumulative doses to circulating blood cells based on predefined blood flow models, organ dose-volume histograms (DVHs), fractionation schedules, and beam delivery times, thereby laying the foundation for establishing more accurate normal tissue complication probability (NTCP) models for severe radiotherapy-induced lymphopenia (SRIL).

In this study, we aimed to apply the HEDOS framework to quantify hematological dose in lung cancer IMRT and IMPT, followed by the development of an NTCP model for predicting SRIL. By integrating patients’ clinical characteristics, radiotherapy parameters, and hematological dose data, this work sought to construct a predictive model capable of accurately estimating the risk of lymphocyte depletion, ultimately providing a theoretical basis for optimizing radiotherapy strategies and improving clinical outcomes in lung cancer patients.

## 2 Materials and methods

### 2.1 Patient cohort and SRIL

We conducted a retrospective analysis of lung cancer patients who underwent radiotherapy at Shandong Cancer Hospital between February 2022 and January 2025. The inclusion criteria were as follows: all patients had pathologically confirmed lung cancer and received curative-intent radiotherapy. Exclusion criteria included: (1) ALC≤ 500/μL prior to radiotherapy; (2) previous history of thoracic irradiation; (3) presence of distant metastasis at diagnosis; (4) incomplete follow-up data; and (5) mixed use of intensity-modulated radiotherapy (IMRT) and intensity-modulated proton therapy (IMPT) during treatment. A total of 131 patients were ultimately enrolled, including 94 in the IMRT group and 37 in the IMPT group. The choice between IMRT and IMPT was determined by a multidisciplinary clinical team based on several factors including tumor stage, tumor location, expected normal tissue sparing, and insurance or resource availability rather than random assignment. All included patients had complete dosimetric and follow-up data. Ethics committee approval was obtained (approval no: 202509105), and the requirement for informed consent was waived due to the retrospective nature of the study. Patient characteristics are summarized in **Table 1**.

**Table 1:** Summary of patient baseline characteristics.

Variable	Photon coherent No SRIL (n=36)	Photon coherent SRIL (n=58)	Photon Total(N=94)	p value	Proton coherent No SRIL(n=25)	Proton coherent SRIL (n=12)	Proton Total(N=37)	p value
Lymphopenia grade, no.								
0	2(5.56)		2(2.13)		8(32)		8(21.62)	
1	9(25)		9(9.57)		6(24)		6(16.22)	
2	25(69.44)		25(26.60)		11(44)		11(29.73)	
3		54(93.10)	54(57.45)			12(100)	12(32.42)	
4		4(6.90)	4(4.26)			0(0)	0(0)	
Sex, no.				0.954				0.304
Male	29(80.56)	47(81.03)	76(80.85)		23(92)	9(75)	32(86.49)	
Female	7(19.44)	11(18.97)	18(19.15)		2(8)	3(25)	5(13.51)	
Age, y	66.5[60-72]	65[58.5-72.25]	66[60-72]	0.717	69[59.5-76]	68.5[51.5-73.25]	69[59-75]	0.363
Pre-RT ALC, 10^3^/μL	1.40[1.29-2.04]	1.47[1.08-1.68]	1.44[1.11-1.77]	0.347	1.64[1.40-2.07]	1.32[1.13-1.61]	1.58[1.16-2]	0.074
Post-RT ALC, 10^3^/μL	0.90[0.66-1.03]	0.78[0.46-1.01]	0.80[0.50-1.0]	0.102	1.15[0.92-1.38]	0.7[0.52-0.94]	0.99[0.81-1.32]	**<0.001**
Prescribed dose per fraction, Gy	2[1.8-2.15]	2[2-2]	2[2-2.15]	0.143	2[2-2.25]	2[2-2]	2[2-2]	0.74
Fraction at last ALC, no.	24.5[19-28]	27.5[25-29]	27[24-29]	**<0.001**	25[17-30]	28[27-31.25]	27[21-30]	0.182
BDT per fraction, s	420[360-452.25]	420[360-480]	420[360-480]	0.812	120[120-180]	180[135-234]	160[120-180]	**0.015**
Total BDT, s	9480[6900-11340]	10800[9540-12960]	10470[9000-12510]	**0.003**	3240[2640-4020]	5370[3735-6552]	3600[2640-5370]	**0.006**
CTV, cc					101.91[36.07-153.70]	182.635[170.81-221.19]	128.27[64.27-186.89]	**0.001**
PTV, cc	132.76[82.82-271.86]	205.43[129.22-289.15]	192.89[94.36-280.02]	0.098				
Lung cancer, no.				0.412				1
SCLC	8(22.22)	9(15.51)	17(18.09)		9(36)	4(33.33)	13(35.14)	
NSCLC	28(77.78)	49(84.49)	77(81.91)		16(64)	8(66.67)	24(64.86)	
Stage, no.				**0.009**				0.476
I/II	9(25)	3(5.17)	12(12.77)		10(40)	3(25)	13(35.14)	
III	27(75)	55(94.83)	82(87.23)		15(60)	9(75)	24(64.86)	
T-stage, no.				0.921				1
T1 or T2	19(52.78)	30(51.72)	49(52.13)		14(56)	6(50)	20(54.05)	
T3 or T4	17(47.22)	28(48.28)	45(47.87)		11(44)	6(50)	17(45.95)	
N-stage, no.				**0.006**				0.256
N0	9(25)	2(3.45)	11(11.70)		8(32)	1(8.33)	9(24.32)	
N1	5(13.89)	4(6.90)	9(9.57)		7(28)	2(16.67)	9(24.32)	
N2	11(30.56)	28(48.28)	39(41.49)		5(20)	4(33.33)	9(24.32)	
N3	11(30.56)	24(41.38)	35(37.23)		6(24)	5(41.67)	11(29.73)	
Chemotherapy, no.				0.873				0.198
Yes	26(72.22)	41(70.69)	67(71.28)		10(40)	8(66.67)	18(48.65)	
No	10(27.78)	17(29.31)	27(28.72)		15(60)	4(33.33)	19(51.35)	

Values are expressed as number of patients (%) or median [interquartile range].

During radiotherapy, ALC was measured weekly. We collected ALC histories and graded RIL according to the most recent ALC recorded during radiotherapy according to the Common Terminology Criteria for Adverse Events (version 5.0). ALC values of 800 to<1000/μL were classified as grade 1, 500 to<800/μL as grade 2, 200 to<500/μL as grade 3, and<200/μL as grade 4. In this study, SRIL was defined as grade ≥3 lymphopenia (ALC<500/μL) occurring during the radiotherapy course.

### 2.2 Treatment delivery

All patients received either IMRT or IMPT. IMRT was delivered by clinical linac including True Beam, Vital Beam, Trilogy etc. IMPT was delivered using the Varian Probeam pencil-beam scanning system. Vacuum cushions and abdominal compression techniques were employed during daily treatments to enhance positional reproducibility.

After CT simulation, CT images were transferred to the Eclipse treatment planning system (TPS) (Varian Medical Systems, Milpitas, CA). For IMRT plans, the gross tumor volume (GTV), encompassing all primary tumors and metastatic regional lymph nodes, was delineated by physicians based on all available clinical information. The planning target volume (PTV) was generated by expanding the GTV with margins of 5–15 mm for nodal regions and 10–20 mm for the primary lesion. For IMPT plans, the clinical target volume (CTV) included the GTV along with subclinical disease, and the planning process incorporated considerations for setup errors (5mm) and range uncertainties (3.5%). A fixed relative biological effectiveness (RBE) value of 1.1 was adopted.

The dose constraints for organs at risk (OARs) were as follows: For the lungs: V_5Gy(RBE)_< 65%, V_20 Gy(RBE)_< 35%, D_mean_< 20 Gy (RBE); for the heart: V_30Gy(RBE)_< 40%, V_40 Gy(RBE)_< 30%; for the esophagus: D_max_< 66 Gy(RBE), V_45Gy(RBE)_< 50%; and for the spinal cord: D_max_< 45 Gy(RBE).All patients were treated with conventional fractionation (1.8-2.15 Gy per fraction, 5 fractions per week), either with radiotherapy alone or concurrent chemoradiotherapy, as detailed in **Table 1**. The chemotherapy regimens adhered to the Chinese Medical Association Lung Cancer Clinical Diagnosis and Treatment Guidelines (2024 edition). For fractionation schemes differing from 2 Gy per fraction, the physical dose was converted to the biologically effective dose (BED) using the linear-quadratic (LQ) model, with an α/β ratio of 10 Gy (31, 32).

### 2.3 Blood DVH paraments

We utilized a pre-generated spatiotemporal blood distribution model as described in Publication 89 of the International Commission on Radiological Protection (33). Under the supervision of clinical physicians, major normal organs including the aorta, bronchi, esophagus, heart, liver, lungs, pulmonary artery and vena cava were contoured slice by slice. DVH derived from these structures served as input parameters for HEDOS. The number of treatment fractions and the corresponding doses included in the analysis were determined based on the last date of ALC measurement during radiotherapy.

### 2.4 NTCP modeling

The Lyman-Kutcher-Burman (LKB) model was employed to establish NTCP models for predicting radiation-induced toxicities (34). The model incorporates the generalized equivalent uniform dose (gEUD) to convert heterogeneous dose distributions into a biologically equivalent uniform dose. The NTCP is mathematically expressed using a probit function:


$$\begin{array}{c} NTCP=\varphi \left(x\right)=\frac{1}{\sqrt{2\pi }}\int_{-\infty }^{x}e^{\left(\frac{-t^{2}}{2}\right)}dt\\ =\frac{1}{2}\left[1+\text{erf}\left(\frac{x}{\sqrt{2}}\right)\right] \end{array}$$

where $x=\frac{gEUD-D_{50}}{m\cdot D_{50}}$ The gEUD is derived from the differential DVH using the power-law relationship:


$$gEUD={\left(\sum_{i}v_{i}D_{i}^{a}\right)}^{\frac{1}{a}}$$

In these equations,*D*_50_ represents the dose corresponding to a 50% complication probability, *m* describes the slope of the dose-response curve, and *a* is the volume effect parameter that reflects the tissue’s architecture and sensitivity to dose heterogeneity (35, 36). The terms *v_i_* and *D_i_* denote the volume fraction and dose value of the *i*th bin of the differential DVH, respectively.

Model parameters were optimized through maximum likelihood estimation (MLE). The negative log-likelihood (NLL) function was minimized using the Nelder-Mead algorithm or an interior-point algorithm, defined as:


$$NLL=-1\cdot \sum_{n}\left[R_{n}\text{log}\varphi \left(x_{n}\right)+\left(1-R_{n}\right)\text{log}\left(1-\varphi \left(x_{n}\right)\right)\right]$$

where *R_n_* represents the binary toxicity outcome for the *n*th patient (e.g., 1 for occurrence of ALC< 500/μL, 0 otherwise).

The robustness of the NTCP model was rigorously assessed. Bootstrap resampling with 1000 iterations was performed to determine the 95% confidence intervals for all fitted parameters. To assess whether the IMRT-derived model could be applied to IMPT patients, the model, originally developed in 94 lung cancer patients receiving photon therapy, was tested in an independent cohort of 37 patients treated with proton therapy.

### 2.5 Statistical analysis

The analyzed clinical and tumor-related parameters encompassed sex, age, tumor volume, T stage and N stage, among others (**Table 1**). Categorical variables were compared using the chi-square test or Fisher’s exact test, while continuous variables were analyzed with the Mann-Whitney U test. A p-value< 0.05 was considered statistically significant. All statistical analyses were performed using SPSS version 27.0 (IBM Corp., Armonk, NY, USA).

Univariate logistic regression analysis (UVA) and multivariate logistic regression analysis (MVA) were conducted to assess the influence of clinical confounding factors on SRIL. In the MVA, we applied Bonferroni correction to adjust p-values. We implemented dummy coding for categorical variables, setting StageI/II (**Table E1**) as the reference category, and dichotomized continuous variables using median thresholds. Furthermore, UVA served to explore correlations between SRIL and blood DVH parameters (D_n%_ and V_nGy_). D_n_% was defined as the minimum dose to the hottest n% volume of an OAR or target, and V_nGy_ as the volume percentage receiving >n Gy. We used analysis of variance (ANOVA) to test for significant differences in mean blood dose among patient groups categorized by RIL grade (≤1, 2, 3, and 4). A chi-square goodness-of-fit test was applied to evaluate the agreement between the observed data and the model predictions. The observed NTCP values were sorted by mean blood dose and divided into five bins. The predicted NTCP values were grouped accordingly into the same bins. A chi-square value closer to zero indicated a better model fit.

The NTCP model was further evaluated using receiver operating characteristic (ROC) curve analysis. The area under the ROC curve (AUC) and the Brier score were calculated to assess the predictive accuracy of the model. Stratified 5-fold cross-validation was implemented to confirm the generalizability of the NTCP model, ensuring that each fold contained a similar proportion of SRIL cases. Model calibration performance was assessed using the Brier score and calibration curve analysis. The observed event rates with 95% confidence intervals across prediction deciles were calculated using the Wilson score method. A smoothed calibration curve was generated using Locally Weighted Scatterplot Smoothing (LOWESS). The calibration slope and intercept were derived by fitting the observed outcomes against the logit-transformed predicted probabilities.

The primary objective of this study was to develop modality-specific NTCP models rather than to estimate the causal effect of treatment modality on SRIL risk. Therefore, propensity score matching or inverse probability weighting was not performed.

## 3.Results

### 3.1 Patient characteristics

A total of 131 patients with lung cancer were included in this study, with detailed baseline characteristics summarized in **Table 1**. SRIL was observed in 61.70% (58/94) of patients receiving IMRT and 32.43% (12/37) of those receiving IMPT. In the IMRT group, the fraction at last ALC measurement, mean blood dose, and blood gEUD were significantly higher in the SRIL group (all p< 0.001). In the IMPT group, the CTV was significantly larger, and the post-radiotherapy ALC was significantly lower in the SRIL group (both p< 0.001). Conversely, no significant differences were found between the groups for a range of other baseline characteristics, including biological sex, age, pre-RT ALC, prescribed dose per fraction, beam delivery time, lung cancer type, T-stage and chemotherapy history (all p > 0.05), in either treatment modality. Dose distributions from randomly selected patients are provided in **Supplementary Figure 1**, which displays the dose maps of in each patient with RIL grade 3. IMRT exhibits a more diffuse low-to-intermediate dose bath, whereas IMPT demonstrates a steeper dose fall-off.

**Figure 1:**
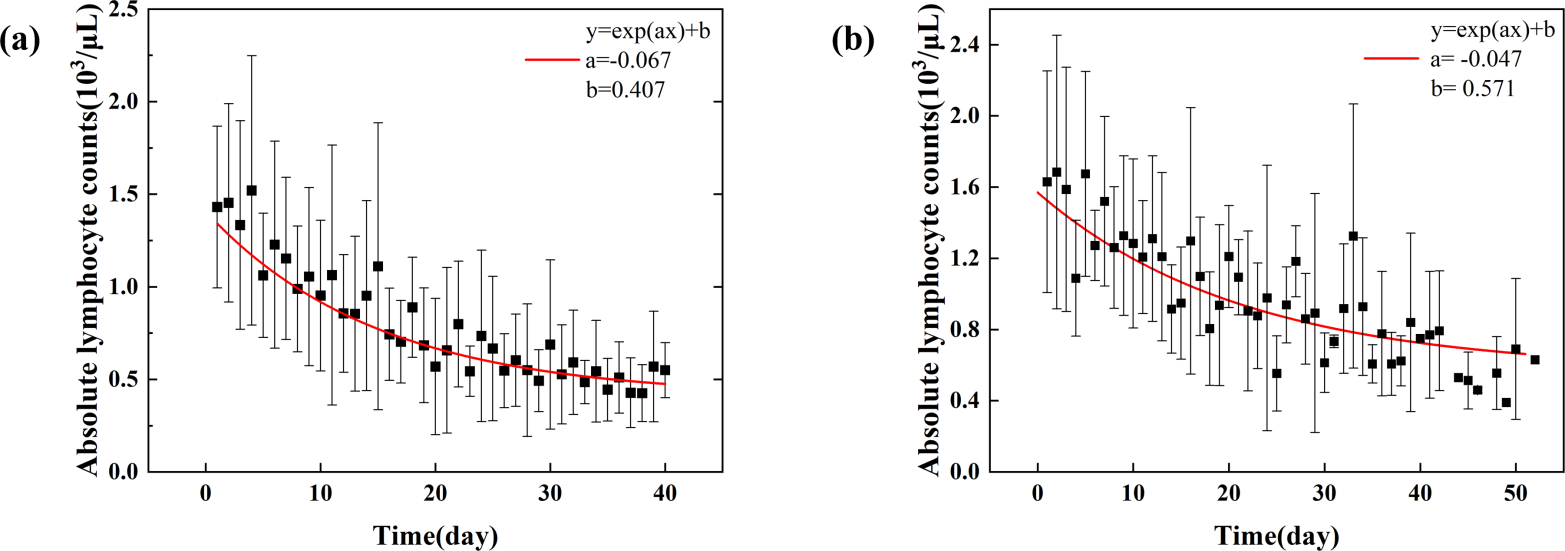
Box plots show the distribution of absolute lymphocyte count (ALC) over time during radiation therapy for patients in the **(A)** photon and **(B)** proton treatment groups.

The average number of ALC measurements per patient during radiotherapy was 6 in the photon group and 5 in the proton group. ALC demonstrated a progressive decline during radiotherapy, and as a result, the average ALC decreased exponentially in both groups, with a daily decay rate of 0.067 for photons and 0.047 for protons, corresponding to an estimated time to half the pre-RT ALC of approximately 10 days and 15 days, respectively (**Figure 1**).

Univariate and multivariate analyses were performed to identify factors associated with SRIL in the photon and proton therapy groups, respectively (**Table E1**). In the photon therapy group, multivariate analysis revealed that advanced tumor stage (Stage III *vs*. Stage I/II; OR = 0.215, 95% CI: 0.051–0.914, p = 0.037) and higher blood gEUD (≥8.18 Gy *vs*.<8.18 Gy; OR = 4.682, 95% CI: 1.774–12.356, p = 0.002) were independently associated with an increased risk of SRIL. In univariate analysis, ‘fraction at last’ of ALC measurement (≥27 *vs*.<27) was also associated with SRIL (OR = 2.600, 95% CI: 1.065–6.348, p = 0.036), though this association was not retained in the multivariate model (p = 0.129). In the proton therapy group, blood gEUD (≥5.05 Gy(RBE) *vs*.<5.05 Gy(RBE)) showed a trend toward association with SRIL in univariate analysis (OR = 4.500, 95% CI: 0.972–20.827, p = 0.054).

**Figure 2:**
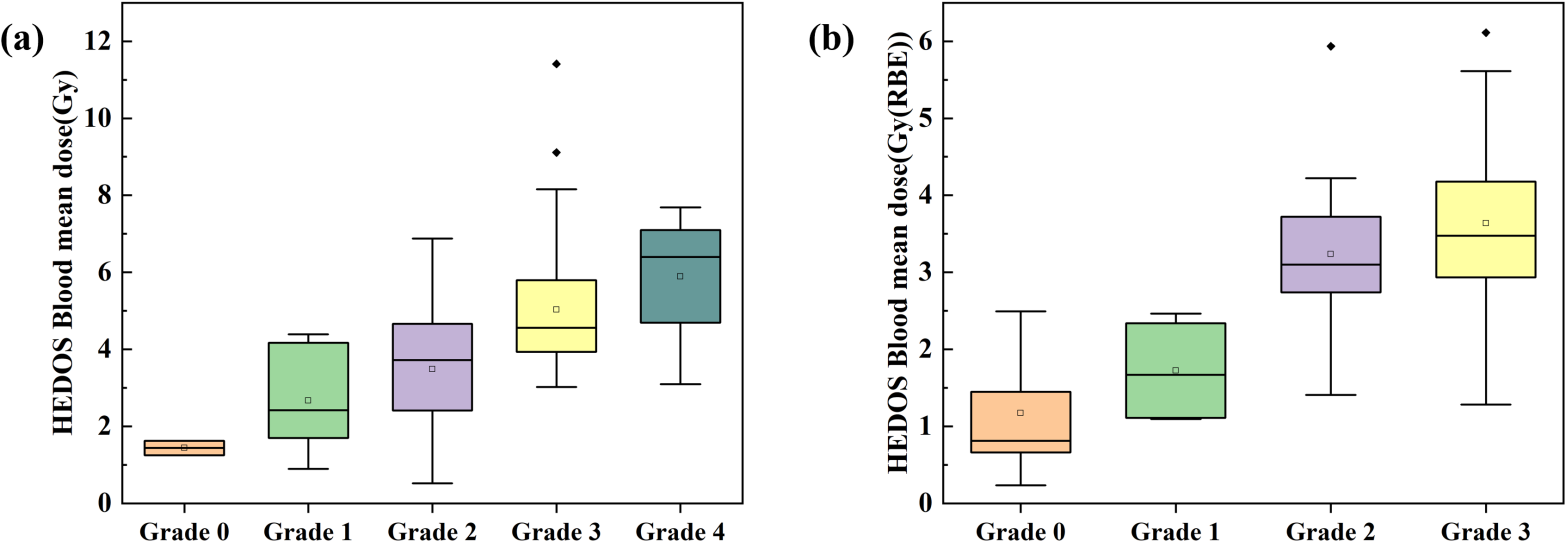
Hematological dose (HEDOS) mean blood dose across lymphopenia grade groups. Box plots show the mean blood dose in patients stratified by lymphopenia grade for the **(A)** photon and **(B)** proton therapy cohorts. The boxes represent the interquartile range (IQR), the solid and dotted lines inside the boxes indicate the median and mean, respectively, and the open squares denote outliers.

Using the HEDOS framework, we calculated the blood DVH, and the detailed characteristics of the DVH parameters are summarized in **Table 2**. The median mean blood dose was 4.17[3.44–5.36] Gy for patients treated with photons and 2.38 Gy(RBE) [IQR: 0.88–3.20] for patients treated with protons. **Figures 2A, B** illustrate the distribution of mean blood doses (mean, median, IQR, and outliers) across patients with RIL grades 0, grade 1, grade 2, grade 3, and grade 4. We observed that patients who developed SRIL received significantly higher mean blood doses in both photon and proton therapy cohorts (ANOVA, p< 0.001). **Table 2** presents the results of the univariable analysis (UVA) of blood DVH parameters. SRIL was associated with each D_n%_ parameter (UVA, OR > 1.005, p< 0.016). In summary, all blood dosimetric parameters demonstrated a significant correlation with the incidence of SRIL.

**Table 2:** Univariate logistic regression analysis for association between blood dose-volume histogram parameters and severe radiation-induced lymphopenia.

Blood DVH paraments	Photon coherent Univariate logistic regression			Proton coherent Univariate logistic regression		
	Median [Interq uartile range]	Odds ratio (95% CIs)	*p*-value	Median [Interquartile range]	Odds ratio (95% CIs)	*p*-value
gEUD, Gy	8.18[6.03-9.95]	1.830(1.422-2.355)	< 0.001	5.05[1.92-6.96]	1.479(1.104-1.981)	0.009
Mean dose, Gy	4.17[3.44-5.36]	2.463(1.626-3.730)	< 0.001	2.38[0.88-3.20]	2.612(1.271-5.366)	0.009
D_10%_, Gy	6.69[5.33-7.97)	2.034(1.497-2.763)	< 0.001	4.03[1.43-5.32]	1.855(1.184-2.906)	0.007
D_20%_, Gy	5.41[4.31-6.91]	2.174(1.540-3.068)	< 0.001	2.86[1.10-3.68]	2.207(1.188-4.099)	0.012
D_30%_, Gy	4.75[3.82-6.11]	2.230(1.548-3.211)	< 0.001	2.42[0.98-3.03]	2.380(1.192-4.751)	0.014
D_40%_, Gy	4.32[3.38-6.62]	2.287(1.561-3.351)	< 0.001	2.07[0.79-2.65]	2.529(1.190-5.377)	0.016
D_50%_, Gy	3.96[3.06-5.20]	2.364(1.582-3.531)	< 0.001	1.83[0.68-2.37]	2.770(1.206-6.364)	0.016
D_60%_, Gy	3.64[2.83-4.84]	2.445(1.605-3.724)	< 0.001	1.62[0.60-2.13]	3.109(1.234-7.833)	0.016
D_70%_, Gy	3.36[2.61-4.50]	2.553(1.637-3.982)	< 0.001	1.43[0.52-1.90]	3.574(1.271-10.049)	0.016
D_80%_, Gy	3.07[2.35-4.17]	2.690(1.676-4.319)	< 0.001	1.21[0.45-1.65]	4.483(1.351-14.882)	0.014
D_90%_, Gy	2.72[2.03-3.70]	2.840(1.693-4.764)	< 0.001	0.93[0.36-1.32]	6.970(1.543-31.488)	0.012
V_1Gy_, %	99.99[99.89-100]	1771.125(9.320-336578.980)	0.005	87.90[24.62-96.44]	1.038(1.006-1.070)	0.019
V_1.5Gy_, %	99.87[98.28-99.99]	1.718(1.184-2.491)	0.004	66.20[9.96-84.74]	1.034(1.007-1.061)	0.012
V_2Gy_, %	98.86[90.83-99.93]	1.113(1.038-1.195)	0.003	42.81[3.86-65.58]	1.035(1.008-1.063)	0.01
V_2.5Gy_, %	94.03[74.25-99.55]	1.005(1.027-1.083)	< 0.001	28.28[1.24-45.02]	1.083(1.007-1.070)	0.016
V_3Gy_, %	82.19[52.48-98.08]	1.042(1.023-1.061)	< 0.001	16.99[0.16-30.55]	1.045(1.005-1.086)	0.025
V_3.5Gy_, %	64.92[37.08-93.95]	1.037(1.021-1.055)	< 0.001	11.57[0.07-22.15]	1.057(1.005-1.113)	0.033

The univariate logistic regression analysis was performed to investigate the association between blood DVH parameters (continuous) and SRIL outcomes (binary). The odds ratio was calculated as the exponential of the coefficient for a parameter in the logistic regression model.

### 3.2 NTCP model

**Figure 3:**
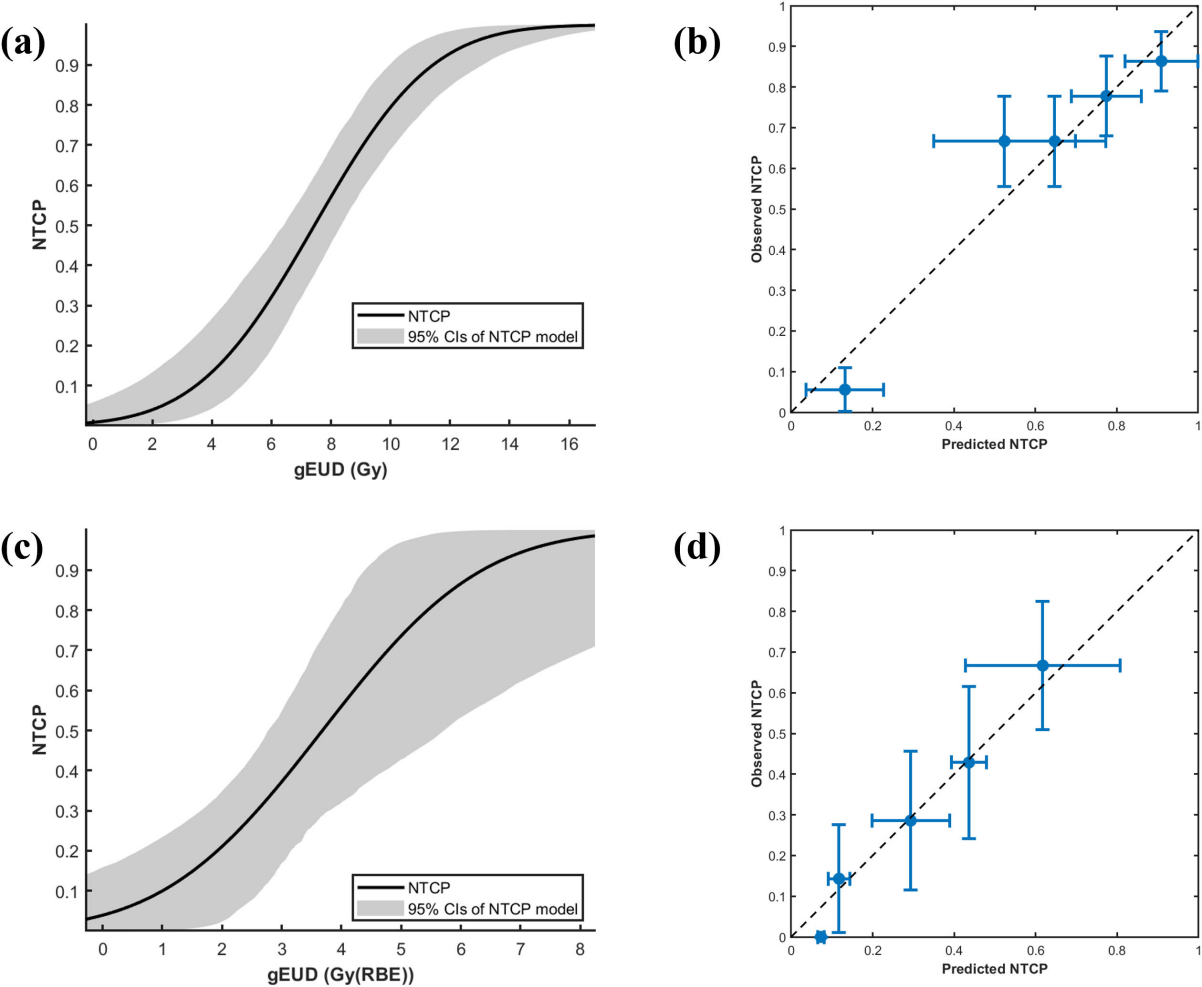
NTCP model parameter fitting and calibration for photon and proton therapy cohorts. **(A)** Parameter estimates with 95% confidence intervals from 1,000 bootstrap iterations for the photon cohort: D_50_ = 7.44 (6.53–8.31) Gy, m = 0.42 (0.28–0.63), a = 19.85 (1.14–29.60). **(B)** Model calibration for photon cohort: solid circles represent mean NTCP values of grouped data with error bars indicating standard deviations; chi-squared test (χ² = 0.09, p = 0.99) indicates good model fit. **(C)** Parameter estimates for the proton cohort: D_50_ = 3.68 (2.74–5.45) Gy(RBE), m = 0.56 (0.26–1.00), a = 2.35 (0.61–14.66). **(D)** Model calibration for proton cohort: solid circles show mean NTCP values with standard deviations; chi-squared test (χ² = 0.08, p = 0.99) confirms good model fit.

Using the maximum likelihood method, we determined the key parameters of the LKB NTCP model for predicting SRIL in patients treated with photon or proton radiotherapy (**Figure 3**).

**Figure 4:**
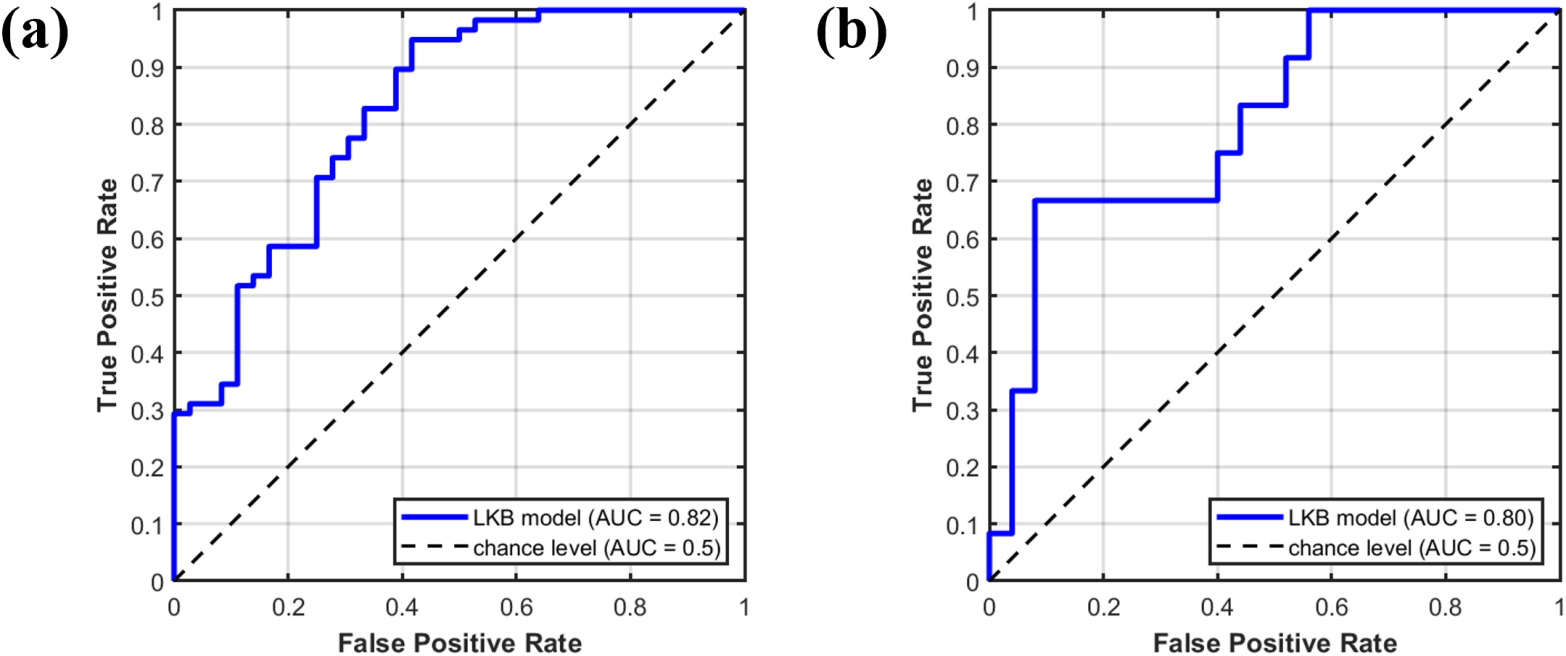
Receiver operating characteristic (ROC) curves for the Lyman-Kutcher-Burman normal tissue complication probability model in predicting severe radiation-induced lymphopenia. **(A)** ROC curve for the photon therapy cohort (AUC = 0.82). **(B)** ROC curve for the proton therapy cohort (AUC = 0.80). The dashed diagonal line represents the reference for a non-informative test (AUC = 0.5).

For the photon cohort (**Figure 3A**), parameter estimates and their 95% confidence intervals were obtained using 1,000 bootstrap iterations. The optimized values of D_50_, m, and a were 7.44 Gy (95% CI: 6.53–8.31 Gy), 0.42 (95% CI: 0.28–0.63), and 19.85 (95% CI: 1.14–29.60), respectively. In **Figure 3B**, solid circles represent the mean NTCP values of grouped data, and error bars indicate the corresponding standard deviations. The chi-squared test comparing grouped observations with predicted SRIL probabilities yielded a value of 0.09 (p = 0.99), indicating good model fit. The ROC curve of the LKB model is shown in **Figure 4A**, with an AUC of 0.82. The Brier score was 0.15. Furthermore, stratified 5-fold cross-validation confirmed the robustness of the model, with an average AUC of 0.81.

For the proton cohort (**Figure 3C**), 1,000 bootstrap iterations yielded optimized parameters with 95% confidence intervals of D_50_ = 3.68 Gy(RBE) (95% CI: 2.74–5.45 Gy(RBE)), m = 0.56 (95% CI: 0.26–1.00), and a = 2.35 (95% CI: 0.61–14.66). In **Figures 3D**, solid circles represent the mean NTCP values of grouped data, with error bars showing the standard deviations. The chi-squared test comparing grouped observations with predicted SRIL probabilities yielded a value of 0.08 (p = 0.99), again indicating good model fit. The ROC curve is shown in **Figures 4B**, with an AUC of 0.80. The Brier score was 0.17. Stratified 5-fold cross-validation further validated model robustness, with an average AUC of 0.74.

**Figure 5:**
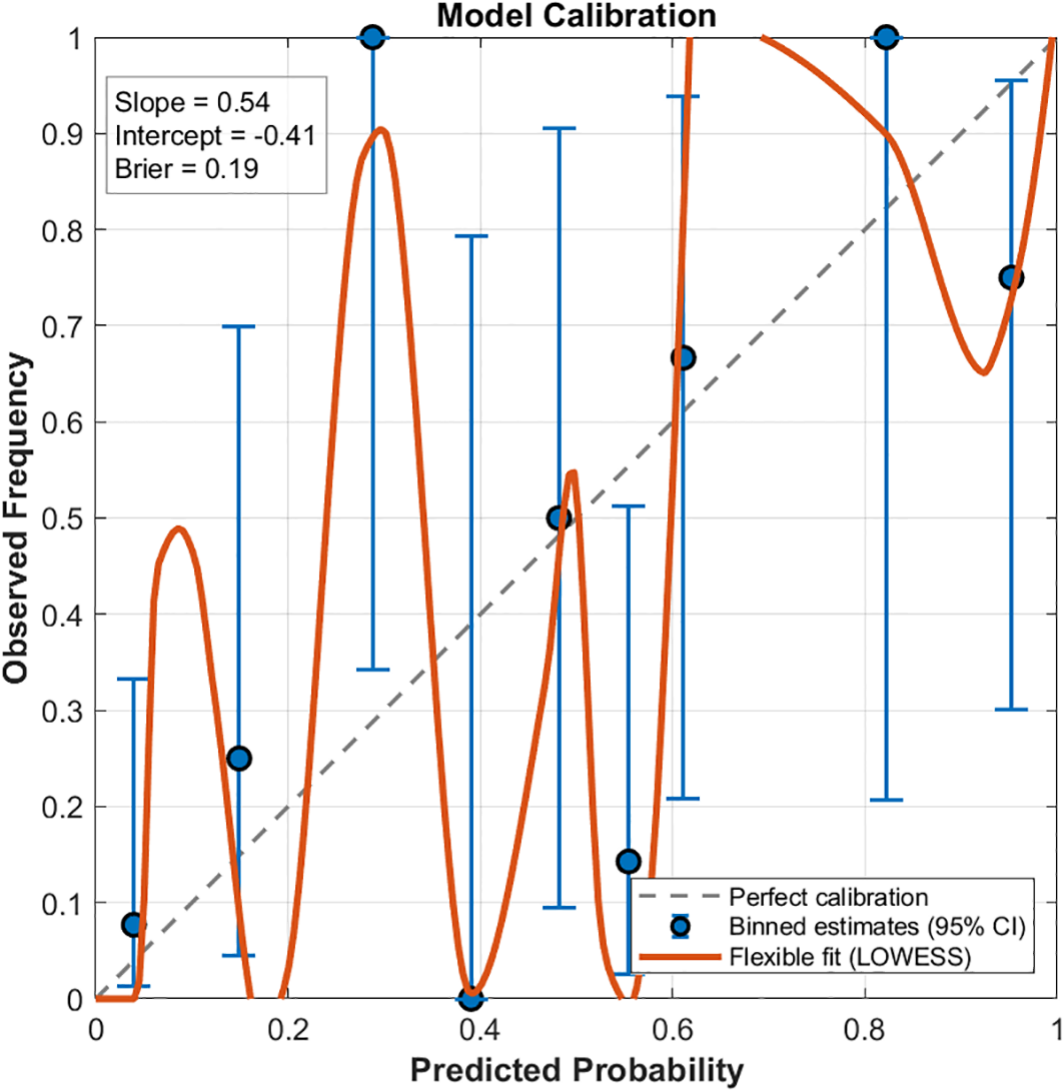
Cross-modality validation of the photon therapy-derived NTCP model in proton therapy patients. The dashed diagonal line indicates perfect calibration. Blue circles with error bars represent observed event rates with 95% confidence intervals across prediction bins. The orange line corresponds to a flexible LOWESS fit showing the smoothed relationship between predicted and observed probabilities.

**Figure 5** displays the cross-modality validation results, illustrating the calibration of the photon therapy-derived NTCP model in a proton therapy cohort. The dashed diagonal line represents perfect calibration, while blue markers with error bars denote the observed event rates (with 95% confidence intervals) across prediction bins. A flexible LOWESS curve (orange line) illustrates the smoothed relationship between predicted and observed probabilities. The dotted horizontal line indicates the overall prevalence of SRIL events in the cohort. Model performance statistics are displayed in the top-left corner, showing a calibration slope of 0.54 and an intercept of -0.41, suggesting that the original model systematically overestimates risk and exhibits underfitting. The Brier score was 0.19, and the discriminative ability was moderate, with an AUC of 0.793. Collectively, these results indicate that although the model distinguishes between patients at different risk levels, its calibration is suboptimal and would benefit from recalibration.

## 4 Discussion

In this study, we developed and validated NTCP models for predicting SRIL in lung cancer patients, based on hematological dose calculations derived from the HEDOS framework. The results demonstrate that blood dose, quantified through HEDOS-based gEUD, is a strong predictor of SRIL in both photon and proton radiotherapy.

Lymphocytes are among the most radiosensitive cell populations in the human body, and previous studies have demonstrated that even low-dose irradiation in the range of 0.125—2 Gy can induce apoptosis or DNA damage (17, 37, 38). In the present study, low-dose parameters such as V_1Gy_ and V_1.5Gy_ were significantly associated with SRIL (**Table 2**), consistent with the concept that the “low-dose bath” contributes meaningfully to lymphocyte depletion. These findings reinforce accumulating evidence that avoiding unnecessary low-dose spillage may be critical for preserving systemic immunity during radiotherapy.

Considering the immune system as an OAR in radiotherapy planning poses dual challenges. On the one hand, immune cells are widely distributed throughout the body, not confined to a specific anatomical structure (39), and are subject to continuous renewal. On the other hand, lymphocytes circulate dynamically through the vascular system, constantly moving in and out of the irradiation field. Notably, previous studies exploring the mechanisms of hematological toxicity have taken two different approaches: some have focused on the dose to fixed organs (e.g., lung, heart) or on global metrics such as whole-body median dose or bone marrow dose (16, 40–42), without fully accounting for temporal dynamics of blood circulation; HEDOS, however, integrates organ-specific DVHs, fractionation timing, and blood-flow models to estimate cumulative exposure of circulating lymphocytes (29, 30). Our study confirms that HEDOS-derived blood gEUD is an independent predictor of SRIL in multivariate analysis, supporting the framework’s clinical utility in thoracic radiotherapy.

Despite the application of strict inclusion and exclusion criteria, residual clinical differences between the IMRT and IMPT cohorts may still exist and warrant careful consideration when interpreting the observed SRIL incidence and NTCP behavior. Imbalances in disease stage and nodal involvement were evident between cohorts (**Table 1**). More advanced disease and higher N-stage—particularly prevalent in the photon cohort—are associated with larger target volumes and more extensive nodal irradiation. This inherently increases the volume of circulating blood exposed to radiation, potentially elevating hematologic dose even when conventional organ-at-risk constraints are met (43, 44). Although concurrent chemotherapy was included as a covariate and was not significantly associated with SRIL in either univariate or multivariable analyses in both cohorts (**Supplementary Table E1**), we acknowledge that chemotherapy may still modulate lymphocyte sensitivity during treatment (45). In addition, beyond the exclusion of severe pre-treatment lymphopenia, baseline immune status differed between cohorts, with the proton cohort exhibiting higher baseline ALC. Baseline ALC has been consistently identified as an important predictor of radiation-induced lymphopenia in prior studies (43, 46, 47), and this imbalance may partially contribute to the lower observed SRIL risk in proton-treated patients. However, despite this baseline advantage, blood-dose distribution differences between modalities appear to play an important role in shaping SRIL risk patterns observed in the models, as evidenced by the distinct NTCP model behavior and calibration observed between photon and proton therapy. Therefore, we caution against interpreting the lower SRIL incidence in the IMPT cohort as direct evidence of proton superiority. Instead, our primary conclusion that modality-specific NTCP models are required due to fundamentally different blood dose distributions remains robust, as it is grounded in dosimetric analysis rather than direct outcome comparison. In addition, the cohort imbalance reflects real-world clinical practice rather than randomized treatment allocation. Future studies with larger and better-balanced cohorts are warranted to further validate these findings.

A central finding of this work is the striking difference in fitted LKB model parameters between photon and proton therapy, which is well supported by the dosimetric data presented in **Table 2** and the model fitting illustrated in **Figure 3** and **5**. Photon-treated patients exhibited significantly higher blood gEUD and mean blood dose (**Table 2**). These differences are further illustrated by a representative comparison of IMRT and IMPT dose distributions (**Supplementary Figure 1**), demonstrating that photon plans produce a more spatially diffuse low-to-intermediate dose bath, whereas proton plans show a sharper dose fall-off. We also observed that the proton D_50_ did not fall below the photon D_50_, and in some scenarios appeared higher, which differs from typical expectations and further suggests that parameter interdependence and dose-range differences may influence model outcomes. To evaluate the stability of these parameter differences, we conducted a constrained-parameter analysis by fixing the volume-effect parameter to the photon-derived value (a = 19.85) and reoptimizing D_50_ and m for the IMPT cohort using maximum likelihood estimation. All goodness-of-fit metrics, including AUC, Brier score, cross-validation AUC, and chi-square calibration tests, were recalculated under this setting. For the proton cohort, fixing the parameter(a) still resulted in an acceptable model fit, with D_50_ = 8.72 Gy(RBE) and m = 0.60. Predictive performance remained adequate (AUC = 0.79; Brier score = 0.17), and the chi-square calibration test again showed excellent agreement between predicted and observed SRIL rates (p = 0.99). Confidence intervals were wider for the proton cohort (D_50_: 6.21–14.98 Gy; m: 0.27–1.10), reflecting the smaller sample size and narrower dose sampling range. Cross-validation AUCs were consistent with the main models (IMPT: 0.79 ± 0.13). Collectively, these results demonstrate that even when constrained to the photon-derived parameter a, the proton cohort can still be fitted successfully, although with different optimal D_50_ and m values, indicating that the observed parameter differences are at least partially driven by dose-range characteristics.

Furthermore, the validation results shown in **Figure 5** demonstrate that the photon-derived model systematically overestimated SRIL risk when applied to the proton cohort, highlighting the limitations of directly transferring NTCP models between modalities. Together, these quantitative differences between photons and protons in both dosimetric parameters (**Table 2**) and fitted model behavior (**Figure 3** and **5**) support the interpretation that the two modalities sample different blood-dose regions and therefore produce distinct optimal parameter sets. These findings indicate that the need for modality-specific NTCP models arises primarily from their differences in dose-distribution characteristics, rather than from intrinsic biological differences in lymphocyte radiosensitivity. Although this study adhered to rigorous inclusion criteria—such as excluding patients with severe baseline lymphopenia and enrolling patients treated at a newly established proton center—these restrictions inevitably resulted in a relatively limited sample size, which may affect the generalizability of our findings. Notably, the NTCP model developed from the photon cohort did not perform optimally when validated in the proton cohort (**Figure 4**). This observation suggests that photon and proton radiotherapy may not share identical dose–response behavior due to their distinct blood-dose distributions. Consequently, we established an independent NTCP model for the proton cohort (**Figure 3C**).

An important translational implication of these findings is that blood-dose exposure cannot be assumed equivalent between modalities, even when target coverage and OAR constraints are similar. Owing to its reduced low-dose bath and lower integral dose, proton therapy may potentially contribute to improved lymphocyte preservation, which is an increasingly relevant consideration for patients receiving concurrent chemotherapy or immunotherapy.

Despite its contributions, this study has several limitations. First, it is retrospective and conducted at a single institution, and the relatively small IMPT sample size—reflecting early implementation at our center—may limit statistical power. Nevertheless, the consistency of NTCP model performance across bootstrap, cross-validation, and calibration analyses indicates that the dose-response relationships identified here are robust. Second, planned rather than delivered dose was used to calculate blood dose. Although beam delivery time differed significantly between patients and is known to influence lymphocyte depletion (30), future studies incorporating real-time dose reconstruction and 4D blood-flow modeling are warranted. Finally, incorporating immunotherapy data would further strengthen clinical relevance, as lymphocyte preservation has direct implications for treatment response and survival.

From a clinical standpoint, the modality-specific SRIL NTCP models developed here could support treatment planning decisions. The planned blood gEUD—calculated via HEDOS—can be input into the appropriate IMRT- or IMPT-based model to estimate individualized SRIL risk. Patients can then be stratified (e.g., low-, intermediate-, high-risk). Those exceeding a defined threshold (e.g., NTCP >30–40%) may benefit from lymphocyte-sparing strategies, such as prioritizing proton therapy, reducing low-dose bath via beam-angle optimization, limiting elective nodal irradiation, or including blood-dose objectives in inverse planning. Low-risk patients can proceed with standard planning. This approach enables proactive balancing of tumor coverage, OAR sparing, and immune preservation.

In summary, hematologic dose is a strong and consistent predictor of SRIL. ·Our results suggest that photon and proton radiotherapy are associated with distinct blood-dose distributions. These differences justify the use of modality-specific models for individualized risk prediction and highlight the potential benefits of dose-optimization or lymphocyte-sparing planning strategies in immuno-oncology.

## 5 Conclusion

Based on DVHs of thoracic and abdominal organs and the calculated blood DVH, this study developed a NTCP model to predict the risk of SRIL during lung cancer radiotherapy. The model demonstrated good fit in their respective cohorts. The blood dose calculated by the HEDOS system showed a correlation with the degree of lymphopenia during radiotherapy. The results indicate that proton and photon radiotherapy techniques produce substantially different blood dose distributions, leading to distinct optimal NTCP model parameters. These differences are primarily attributable to the modalities’ dose-delivery characteristics rather than reflecting fundamental biological differences in lymphocyte injury mechanisms. Therefore, lymphocyte injury models established based on photon radiotherapy should not be directly applied to risk assessment in proton radiotherapy. Developing modality-specific NTCP models is essential to improve individualized toxicity prediction and clinical decision-making.

## Data Availability

The raw data supporting the conclusions of this article will be made available by the authors, without undue reservation.
