# Supplementary material for: Normal tissue complication probability modeling of severe radiation-induced lymphopenia using blood dose for lung cancer patients treated with IMRT and IMPT
Source: Front Immunol. 2026 Apr 7;17:1752785. doi: 10.3389/fimmu.2026.1752785 (PMC13095790; doi:10.3389/fimmu.2026.1752785)

Supplementary Material

**Supplementary Table E1** Logistic regression analysis for factors associated with severe radiation-induced lymphopenia.

| **Photon coherent** |  | | | | | | | | **Proton coherent** |  | | | | | | | |
| --- | --- | --- | --- | --- | --- | --- | --- | --- | --- | --- | --- | --- | --- | --- | --- | --- | --- |
| variable | Univariate logistic regression | | | | Multivariable logistic regression | | | | variable | Univariate logistic regression | | | | Multivariable logistic regression | | | |
|  | Odds ratio | 95% CI | | p value |  | 95% CI | | p value |  | Odds ratio | 95% CI | | p value |  | 95% CI | | p value |
|  |  | Lower | Upper |  | Odds ratio | Lower | Upper |  |  |  | Lower | Upper |  | Odds ratio | Lower | Upper |  |
| sex |  |  |  |  |  |  |  |  | sex |  |  |  |  |  |  |  |  |
| Male vs female | 1.031 | 0.359 | 2.961 | 0.954 |  |  |  |  | Male vs female | 0.261 | 0.037 | 1.830 | 0.176 |  |  |  |  |
| Age, y |  |  |  |  |  |  |  |  | Age, y |  |  |  |  |  |  |  |  |
| ≥66 vs＜66 | 0.813 | 0.353 | 1.870 | 0.625 |  |  |  |  | ≥69 vs <69 | 0.774 | 0.193 | 3.107 | 0.718 |  |  |  |  |
| Pre-RT ALC, 10^3^/μL |  |  |  |  |  |  |  |  | Pre-RT ALC, 10^3^/μL |  |  |  |  |  |  |  |  |
| ≥1.44 vs ＜1.44 | 1.197 | 0.521 | 2.754 | 0.671 |  |  |  |  | ≥1.58 vs <1.58 | 0.222 | 0.048 | 1.028 | 0.054 |  |  |  |  |
| Prescribed dose per fraction, Gy |  |  |  |  |  |  |  |  | Prescribed dose per fraction, Gy(RBE) |  |  |  |  |  |  |  |  |
| ≥2 vs＜2 | 0.003 | 0.230 | 0.087 | 0.605 |  |  |  |  | ≥2 vs <2 | 0.500 | 0.108 | 2.314 | 0.375 |  |  |  |  |
| Fraction at last ALC, no. |  |  |  |  |  |  |  |  | Fraction at last ALC, no. |  |  |  |  |  |  |  |  |
| ≥27 vs＜27 | 2.600 | 1.065 | 6.348 | **0.036** | 2.169 | 0.799 | 5.888 | 0.129 | ≥27 vs <27 | 3.000 | 0.709 | 12.694 | 0.136 |  |  |  |  |
| BDT per fraction, s |  |  |  |  |  |  |  |  | BDT per fraction, s |  |  |  |  |  |  |  |  |
| ≥420 vs <420 | 1.579 | 0.624 | 3.996 | 0.335 |  |  |  |  | ≥160 vs <160 | 5.333 | 1.142 | 24.899 | **0.033** | 12.336 | 0.785 | 193.726 | 0.074 |
| Total BDT, s |  |  |  |  |  |  |  |  | Total BDT, s |  |  |  |  |  |  |  |  |
| ≥10470 vs <10470 | 2.074 | 0.888 | 4.843 | 0.092 |  |  |  |  | ≥3600 vs <3600 | 7.714 | 1.603 | 37.134 | **0.011** | 1.974 | 0.223 | 17.445 | 0.541 |
| PTV, cc |  |  |  |  |  |  |  |  | CTV, cc |  |  |  |  |  |  |  |  |
| ≥192.89 vs <192.89 | 1.723 | 0.743 | 3.994 | 0.205 |  |  |  |  | ≥128.27 vs <128.27 | 10.625 | 1.874 | 60.246 | **0.008** | 25.316 | 1.913 | 334.963 | 0.014 |
| Stage |  |  |  |  |  |  |  |  | Stage |  |  |  |  |  |  |  |  |
| I/II vs III | 0.164 | 0.041 | 0.654 | **0.010** | 0.215 | 0.051 | 0.914 | 0.037 | I/II vs III | 0.500 | 0.108 | 2.314 | 0.375 |  |  |  |  |
| Chemotherapy |  |  |  |  |  |  |  |  | Chemotherapy |  |  |  |  |  |  |  |  |
| No vs yes | 0.928 | 0.369 | 2.334 | 0.873 |  |  |  |  | No vs yes | 1.063 | 0.555 | 2.039 | 0.853 |  |  |  |  |
| Blood gEUD, Gy |  |  |  |  |  |  |  |  | Blood gEUD, Gy(RBE) |  |  |  |  |  |  |  |  |
| ≥8.18 vs <8.18 | 5.700 | 2.252 | 14.426 | **＜0.001** | 4.682 | 1.774 | 12.356 | 0.002 | ≥5.05 vs <5.05 | 4.500 | 0.972 | 20.827 | 0.054 |  |  |  |  |

**Supplementary Figure 1** Absolute dose distributions in patients with grade 3 radiation-induced lymphopenia: (a) photon radiotherapy and (b) proton radiotherapy.


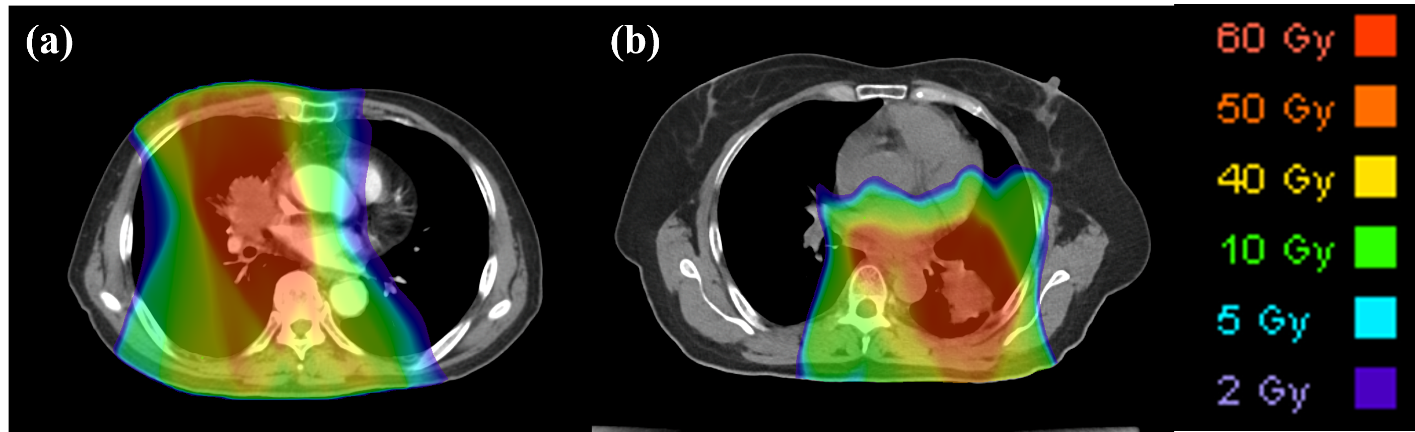

Supplement: Supplementary file 2 [file Table2.docx]
